# Analogs of imine resveratrol alleviate oxidative stress‐induced neurotoxicity in PC12 cells via activation of Nrf2

**DOI:** 10.1002/2211-5463.13209

**Published:** 2021-07-02

**Authors:** Yin Zhang, Zhixiong Wang, Jiehong Yang, Yu He, Haitong Wan, Chang Li

**Affiliations:** ^1^ Zhejiang Chinese Medical University Hangzhou China

**Keywords:** apoptosis, imine resveratrol analog, Nrf2, oxidative stress

## Abstract

Oxidative stress is closely associated with neurodegenerative, cardiovascular and metabolic diseases. Resveratrol and related compounds have shown great potential as antioxidants via either direct scavenging of abundant reactive oxygen species (ROS) or activation of the Kelch‐like ECH‐associated protein 1‐nuclear factor (erythroid‐derived 2)‐like 2‐antioxidant response elements pathway. In the present study, we evaluated imine resveratrol analogs (IRAs) for their neuroprotective effects against ROS in PC12 cells, which are a commonly employed model system for studies of neuronal development and function. We identified that IRA‐3 (4‐[[(4‐hydroxyphenyl)methylene]amino]‐phenol) was more potent than resveratrol at rescuing PC12 cells from H_2_O_2_‐induced oxidative damage, exhibiting a recovery percentage of 60.4% at 50 μm. Our findings suggest that the neuroprotective effect of IRA‐3 was achieved via multiple routes, including direct scavenging of ROS, rescue of endogenous antioxidants and activation of the Kelch‐like ECH‐associated protein 1‐nuclear factor (erythroid‐derived 2)‐like 2‐antioxidant response elements pathway. Our results suggest that IRA‐3 may have potential for development into a possible treatment for neurodegenerative diseases.

## Graphical abstract

Fourteen imine resveratrol analogs were screened for their neuroprotective activity in H_2_O_2_‐treated PC12 cell models. Imine resveratrol analog‐3 was selected as most potent compound with a recovery percentage of 60.4% at 50 μm, which was considerably more potent than resveratrol and edaravone. The neuroprotective effect was exerted via direct scavenging of reactive oxygen species, rescue of endogenous antioxidants and activation of the nuclear factor (erythroid‐derived 2)‐like 2‐antioxidant response elements pathway.

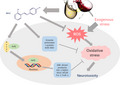

**Abbreviations**
ARE: antioxidant response elements
CCK‐8: cell counting kit‐8
DPPH: 1,1‐diphenyl‐2‐picrylhydrazyl
GCLC: glutamate‐cysteine ligase catalytic subunit
GCLM: glutamate‐cysteine ligase modifier subunit
GSH: glutathione
HO‐1: heme oxygenase‐1
IRAs: imine resveratrol analogs
Keap1: Kelch‐like ECH‐associated protein 1
LDH: lactate dehydrogenase
MDA: malondialdehyde
NQO‐1: NAD(P)H quinone dehydrogenase
Nrf2: nuclear factor (erythroid‐derived 2)‐like 2
PBS: phosphate‐buffered saline
ROS: reactive oxygen species
RT‐PCR: reverse transcriptase‐PCR
SOD: superoxide dismutase
Trx‐1: thioredoxin‐1
TrxR‐1: thioredoxin reductase 1

Oxidative stress is usually caused by the generation and accumulation of reactive oxygen species (ROS), which are produced from both the activation of endogenous metabolic pathways and exogenous stimulation [1]. Many studies have indicated that oxidative stress is closely associated with most neurodegenerative diseases, cardiovascular diseases and metabolic diseases [2, 3, 4]. At the cellular level, ROS can induce cell apoptosis via peroxidative damage to DNA, lipids and proteins [5]. The elimination of ROS from cells depends on both exogenous antioxidants (e.g. vitamin C and curcumin) and the cellular antioxidative defense system, which includes endogenous antioxidants, such as glutathione (GSH) and cytoprotective proteins. The nuclear factor (erythroid‐derived 2)‐like 2 (Nrf2)‐ antioxidant response elements (ARE) pathway is one of the most crucial pathways in regulating antioxidative responses in mammalian cells [6, 7, 8, 9]. In recent years, much effort has been devoted to studying the activation of Nrf2, which occurs mostly through the covalently modification of Kelch‐like ECH‐associated protein 1 (Keap1), an inhibitor of Nrf2 [10, 11, 12]. The development of natural product inspired synthetic analogs is included among the effective strategies in searching of Nrf2 activators [13, 14].

In previous studies, resveratrol and related natural compounds have shown great potential as antioxidants via either direct scavenging of abundant ROS or activation of the Keap1‐Nrf2‐ARE pathway [7]. For example, resveratrol has been reported to suppress oxidative stress and the inflammatory response in diethylnitrosamine‐initiated rat hepatocarcinogenesis [15]. Pallidol, a naturally occurring resveratrol dimer, was also confirmed to be a bi‐functional antioxidant with strong singlet oxygen scavenging ability and moderate Nrf2 inducing activity [16].

A few different series of resveratrol derivatives and analogs have also been designed to improve the bioavailability and enhance the protective effect of resveratrol related compounds, enlightened by the promising results reported for resveratrol [17]. In our previous studies, a series of easily accessible resveratrol analogs, imine resveratrol analogs (IRAs), were designed and synthesized, replacing the C=C double bond of resveratrol with a C=N double bond. Luciferase assays conducted in association with quantitative structure–activity relationship studies helped with the understanding and optimization of several 6‐OH IRAs as enhanced Nrf2 activators [18]. In addition, 6‐OH IRAs have also been reported to have strong quenching activities against 1,1‐diphenyl‐2‐picrylhydrazyl radical, singlet oxygen (^1^O_2_), hydroxyl radical (OH) and super peroxide anion ($O_{2}^{‐}$) [19]. Very recently, 2‐methoxyl‐3,6‐dihydroxyl‐IRA was demonstrated to ameliorate colitis through the activation of Nrf2 and the inhibition of NACHT, LRR and PYD domains‐containing protein 3 expression both *in vivo* and *in vitro* [20]. In the present study, as part of a continuous effort to explore the biological benefits of IRAs, the protective effects of IRAs were investigated in neural cell models. In total, in the rat pheochromocytoma PC12 cell line, which has become a commonly employed model system for studies of neuronal development and function science [21, 22], 14 IRAs were screened for their protective effects against H_2_O_2_‐induced oxidative damage. Among them, IRA‐3 showed the most significant neuroprotective effect. Further pharmacological studies confirmed that Nrf2 activation is crucial in the protective process.

## Results and Discussion

### Synthesis and cytotoxic effects of IRAs

**Fig. 1:**
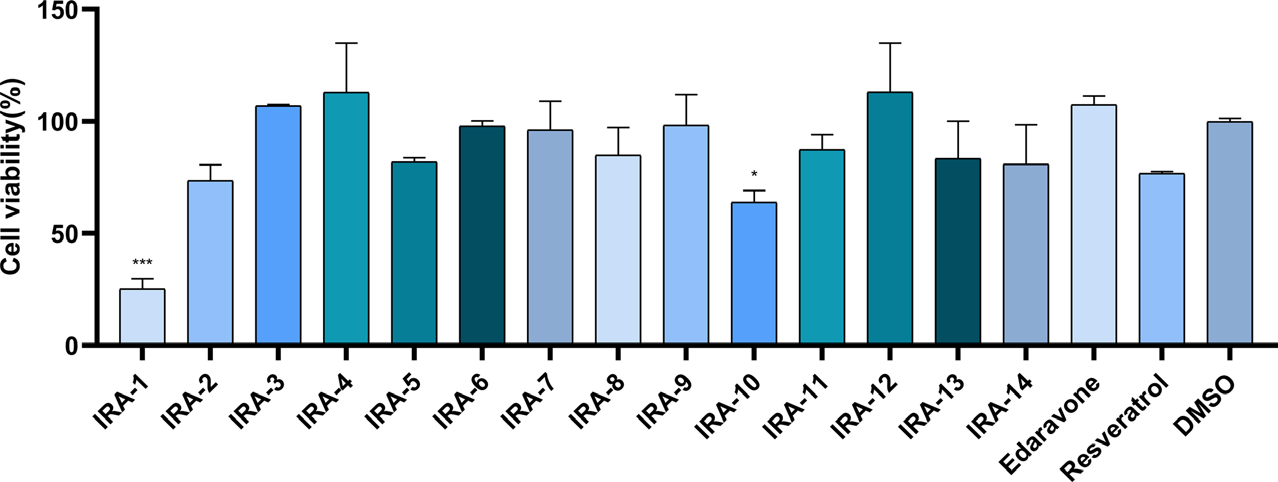
Cytotoxicity of IRAs to PC12 cells. Cells were incubated with 100 μm of one of the IRAs, resveratrol, edaravone or control (serum‐containing medium with 0.1% dimethylsulfoxide) for 24 h, and then cell viability was determined using a CCK‐8 assay. Results are presented as the mean ± SD (*n* = 4). *P < 0.05; ***P < 0.001 vs. the control group. Data were analyzed using one‐way analysis of variance.

In total, 14 IRAs (Table 1) were synthesized using the same procedure as that described previously [18, 19]. The cytotoxicity of each compound at a concentration of 100 μm to PC12 cells was then determined using a CCK‐8 assay. As shown in Fig. 1, all IRAs except IRA‐1 exhibited low toxicity to PC12 cells, with cell viability exceeding 80%. However, 6‐OH substituted IRA‐1 was significantly toxic to PC12 cells, in contrast to its effects on other cells. This phenomenon indicated that, although 6‐OH IRAs have been optimized as a potent chemopreventors for lung cancer, they might not suitable for neurologic system. It is noteworthy that twelve IRAs exhibited lower cytotoxicity than resveratrol at 100 μm.

**Table 1:** Chemical structures of the IRAs.

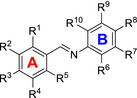							
No.	Ring A				Ring B		
	R^1^	R^2^	R^3^	R^4^	R^6^	R^7^	R^8^
IRA‐1	H	H	H	H	OH	H	H
IRA‐2	OH	H	H	H	H	OH	H
IRA‐3	OH	H	H	H	H	H	OH
IRA‐4	H	H	OH	H	H	H	OH
IRA‐5	H	OMe	H	OMe	H	H	OH
IRA‐6	H	OH	OH	H	H	H	OH
IRA‐7	H	H	OH	H	Me	H	H
IRA‐8	H	OMe	H	H	Me	H	H
IRA‐9	H	OMe	H	H	H	Me	H
IRA‐10	H	H	OH	H	H	Me	H
IRA‐11	H	OMe	H	H	H	H	Me
IRA‐12	H	H	Cl	H	H	H	Me
IRA‐13	H	H	OMe	H	H	H	OMe
IRA‐14	H	H	OMe	H	OH	H	H

### Rescue of H_2_O_2_‐induced oxidative damage by IRAs

**Fig. 2:**
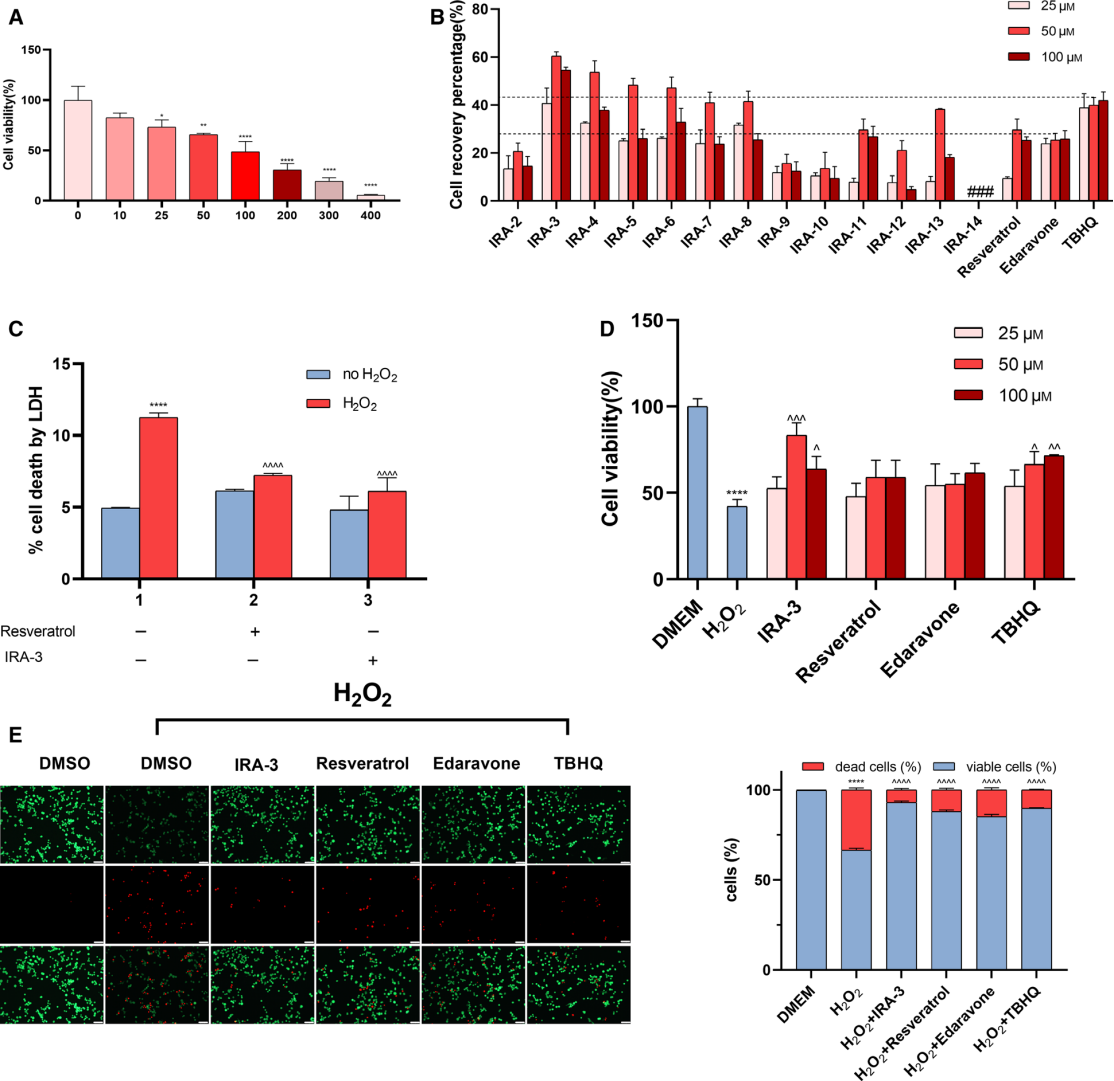
Effects of IRAs on H_2_O_2_‐induced cell damage in PC12 cells. Cells (1 × 10^4^ cells per well) were treated with 0, 10, 25, 50, 100, 200, 300 or 400 μm H_2_O_2_ for 6 h, and then cell viability was then determined using a CCK‐8 assay (A). Cells were treated with 100 μm H_2_O_2_ for 6 h, then 25, 50 or 100 μm IRAs, resveratrol, edaravone or TBHQ was added. After incubation for 24 h, a CCK‐8 assay was used to measure cell recovery percentage (B). The LDH activity in the culture medium was measured using an LDH assay kit (C), and a Calcein AM Cell Viability Assay Kit (D), Calcein AM and PI staining (E) were employed to determine the cell viability. Scale bars = 50 μm. Cell recovery percentage was calculated as described in the Materials and methods (B). PC12 cells were treated with 100 μm H_2_O_2_ for 6 h and then with 50 μm IRA‐3 or resveratrol for another 24 h. The LDH activity in the culture medium was measured using an LDH assay kit (C). The results are presented as the mean ± SD (*n* = 4). *P < 0.05; **P < 0.01;****P < 0.0001 vs. the control group. ^^^ *P* 0.05; ^^^^ *P* < 0.01; ^^^^^ *P* < 0.001; ^^^^^^ *P* < 0.0001 vs. the H_2_O_2_ treatment. ^###^Cell recovery percentage of IRA‐14 (25 μm, −14.6%; 50 μm, −89.5%; 100 μm, −149.4%). Data were analyzed using one‐way analysis of variance (A) and two‐way analysis of variance (C–E).

Aiming to investigate the cytoprotective functions of IRAs, the antioxidative effects of IRAs were evaluated in PC12 cell models [23]. H_2_O_2_ is a universal oxidant and widely used to generate oxidative stress models, including those involving PC12 cells [24]. In the present study, the suitable concentration of H_2_O_2_ was first optimized to establish a stable model. As shown in Fig. 2A, the treatment of PC12 cells with 100 μm H_2_O_2_ for 6 h led to the death of approximately 50% of cells. Thus, this concentration was employed as the model condition. In screening of neuroprotective effects of the IRAs, the cell recovery percentage (for the calculation method, see the Materials and methods) was introduced to more clearly determine the effects of the IRAs. As shown in Fig. 2B, in the presence of 25, 50 and 100 μm of IRAs (except IRA‐14), H_2_O_2_‐treated PC12 cells could recover from oxidative damage. IRAs showed a dose‐dependent manner, with the 50 μm dose being associated with the highest recovery capacity for most IRAs. From the view of a structure–activity relationship, it could be observed that the substitution on Ring B is more crucial. IRAs with R^8^ as a hydroxyl group (IRA‐3, 4, 5 and 6) showed better neuroprotectivities with cell recovery percentages over 40% at 50 μm. 6‐Methyl IRAs (IRA‐7 and 8) are also moderate neuroprotectors. Meanwhile, 7‐methyl IRAs showed no significant capacities in protecting PC12 cells against oxidative stress. Among the screened IRAs, IRA‐3 was selected as the best candidate for further investigation, with a recovery percentage of 60.4% at 50 μm, which was much higher than that of edaravone (25.4%, 50 μm), TBHQ (40.0%, 50 μm) or resveratrol (29.7%, 50 μm). Edaravone is an approved brain‐protective agent for the treatment of ischemic stroke in Japan, China and other Asian counties [25]. TBHQ is a typical antioxidant, scavenging ROS indirectly [26]. Calcein AM release and LDH leakage content in the culture medium was then determined to confirm the CCK‐8 results with IRA‐3 (Fig. 2C–E).

### Alleviation of H_2_O_2_‐induced apoptosis by IRA‐3 treatment

**Fig. 3:**
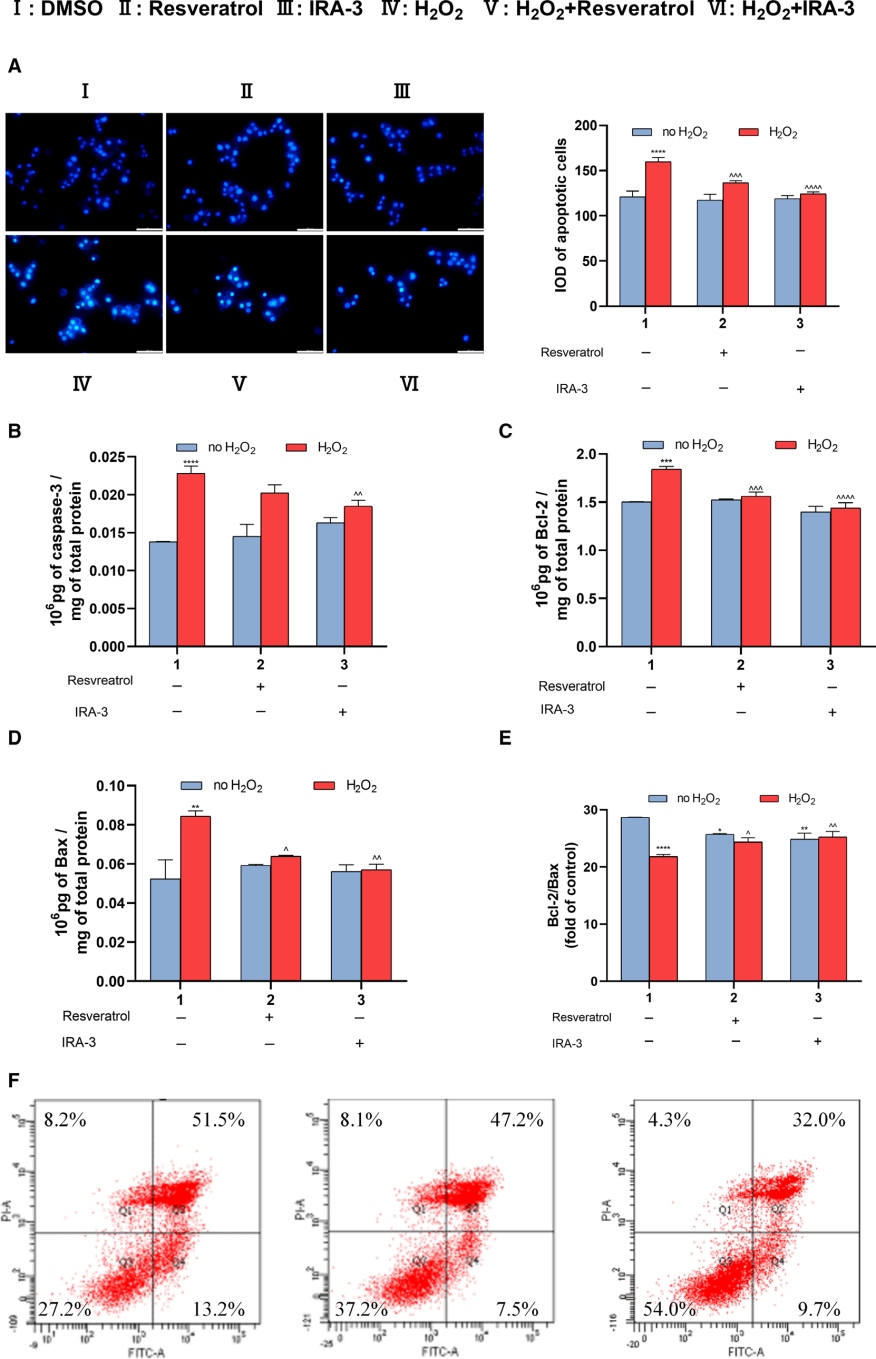
Effects of IRA‐3 on apoptosis of PC12 cells treated with H_2_O_2_ and IRA‐3. Cells were treated with 0 or 100 μm H_2_O_2_ for 6 h, then 50 μm resveratrol or IRA‐3 was added. After being incubated for 24 h, apoptotic cells were measured via Hoechst 33342 staining. Scale bars = 50 μm (A). Expression of apoptosis‐related proteins (caspase‐3, Bcl‐2, Bax, Bcl‐2/Bax) was determined by ELISA (B–E). Flow cytometry of annexin/PI double staining for H_2_O_2_‐treated only, and resveratrol or IRA‐3 and H_2_O_2_‐treated PC12 cells (F). The results are presented as the mean ± SD (*n* = 4). **P < 0.01; ****P < 0.0001 vs. the control group. ^^^ *P* < 0.05; ^^^^ *P* < 0.01; ^^^^^ *P* < 0.001 vs. the H_2_O_2_ treatment. Data were analyzed using two‐way analysis of variance.

Apoptosis is closely associated with in oxidative stress‐induced cell damage. In addition to the PI staining experiment described in Fig. 2E, the population of apoptotic PC12 cells was also determined using fluorescence microscopy after Hoechst 33342 staining [24, 27]. As shown in Fig. 3A, most cells in the control group were viable (I), whereas exposure to H_2_O_2_ resulted in a significant increase in the number of apoptotic cells, as indicated by blue fluorescence (IV). The nucleus was found to bedensely stained, or fragmented densely stained and condensed in a half‐moon shape. Treatment of PC12 cells with 50 μm resveratrol (II) or IRA‐3 (III) also induced cell apoptosis to some level. After H_2_O_2_ treatment, damaged cells were incubated with resveratrol (V) or IRA‐3 (VI), and the percentage of apoptotic cells decreased significantly to various degrees. IRA‐3 exerted a stronger anti‐apoptosis effect than resveratrol. After cell apoptosis, the expression levels of Bcl‐2, Bax [28], cytochrome *c* [29, 30], caspase‐3, Bim, caspase‐9, cleaved PARP [31] and other proteins were varied. The results were further confirmed by the measurement of apoptosis‐related protein expression by an ELISA (Fig. 3B–E). The changes of the levels of caspase‐3 (standard curve equation: *y* = 0.0028*x* − 0.0017, *r* ^2^ = 0.9993), Bax (standard curve equation: *y* = 0.0317*x* + 0.0043, *r* ^2^ = 0.9992) and Bcl‐2 (standard curve equation: *y* = 0.0021*x* + 0.0093, *r* ^2^ = 0.9987) activity were observed in the same manner. Exposure to H_2_O_2_ led to significant increases in the levels of these indicators, whereas the usage of resveratrol and IRA‐3 down‐regulated the levels of all three apoptosis‐related proteins. Notably, IRA‐3 showed better effects than resveratrol in all of the ELISA assays and fluorescence intensity determinations, together with lower apoptosis‐inducing effect than resveratrol in PC12 cells without pretreatment.

To further confirm the anti‐apoptotic effects of IRA‐3, annexin V/PI double staining and flow cytometry analysis were performed to quantify early (annexin V^+^/PI^−^) or late apoptotic (annexin V^+^/PI^+^) cells. The representative images for flow cytometry and the summarized data are presented in Fig. 3F. After H_2_O_2_ treatment, the ratio of early apoptosis increased to 13.2% but stayed in relative low level in IRA‐3 (9.7%) or resveratrol (7.5%) treated group. Consistently, after exposure to H_2_O_2_, the ratio of late apoptotic cells increased to 51.5%, while treatment with IRA‐3 reduced the percentage of late apoptosis to 32.0%, which is more significant than the resveratrol group (47.2%).

### Inhibition of ROS accumulation by and antioxidative effect of IRA‐3

**Fig. 4:**
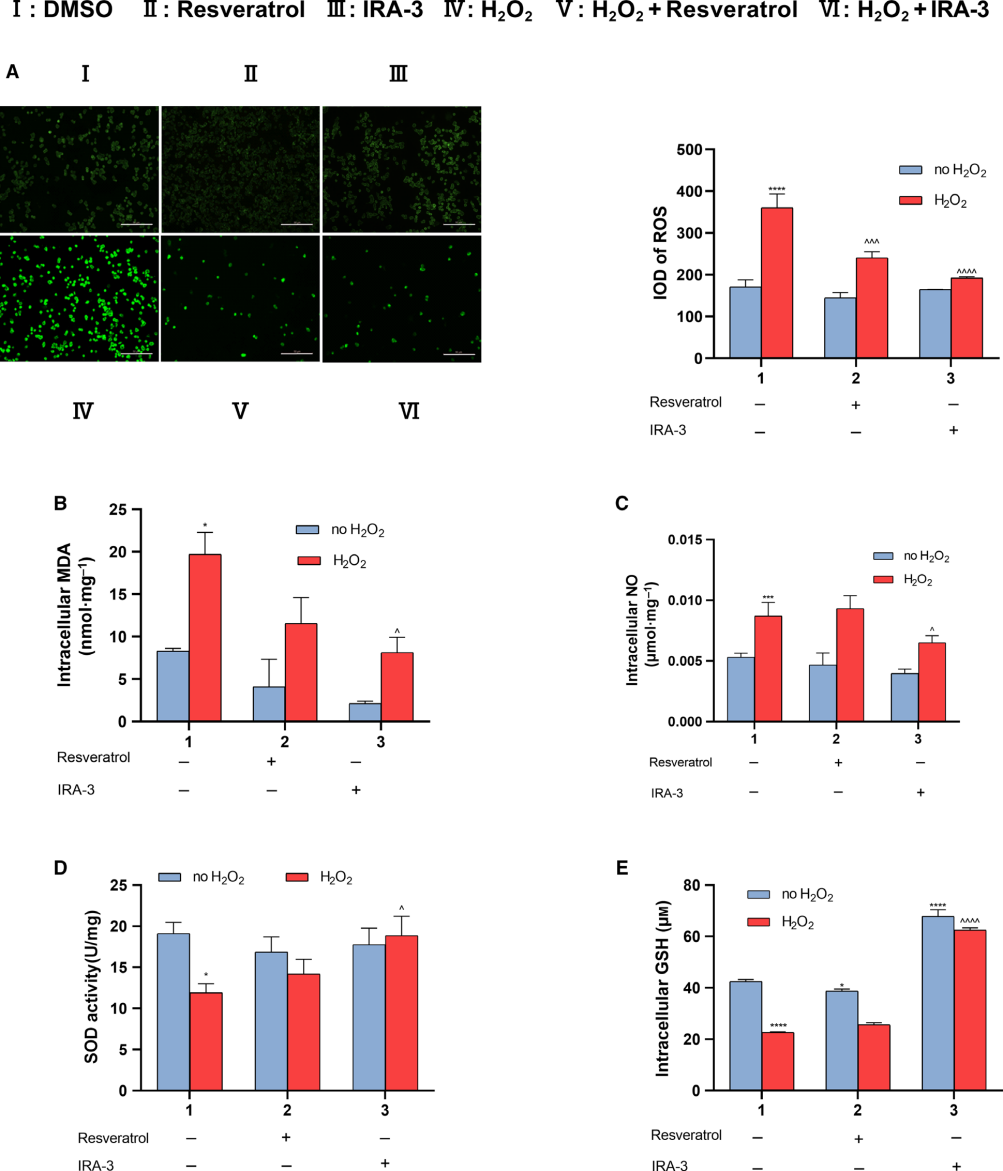
Effects of IRA‐3 on ROS accumulation in normal and model PC12 cells. Cells were treated with 0 or 100 μm H_2_O_2_ for 6 h, and then 50 μm of resveratrol or IRA‐3 was added. After incubation for 24 h, intracellular ROS level was monitored using a DCFH‐DA fluorescent probe. Scale bars = 50 μm (A). The intracellular MDA (B), NO (C), SOD (D) and GSH levels (E) were measured using assay kits. The results are presented as the mean ± SD (*n* = 4). *P < 0.05; ***P < 0.001; ****P < 0.0001 vs. the control group. ^^^ *P* < 0.05; ^^^^^ *P* < 0.001; ^^^^^^ *P* < 0.0001 vs. H_2_O_2_ treatment. Data were analyzed using two‐way analysis of variance.

Previous studies have indicated that antioxidants often achieve their neuroprotective activity by lowering ROS contents in cells. Therefore, we tested the effect of IRA‐3 on H_2_O_2_‐induced oxidative stress in PC12 cells. The production and accumulation of intracellular ROS was monitored using DCFH‐DA, a fluorescent probe employed for ROS detection. Strong increases in ROS level were observed after exposure to H_2_O_2_ (I and IV) (Fig. 4A). The treatment of model PC12 cells with resveratrol or IRA‐3 significantly decreased ROS level (V and VI) (Fig. 4A), whereas the treatment compounds had no effects on ROS in normal PC12 cells (II and III) (Fig. 4A). H_2_O_2_ treatment can not only directly damage cells but also cause the up‐regulation of a series of oxidative stress‐related small molecules. The levels of intracellular malondialdehyde (MDA) and NO were determined by corresponding assay kits. As shown in Fig. 4B,C, the levels of both MDA and NO were up‐regulated by H_2_O_2_ treatment and down‐regulated by resveratrol and IRA‐3 to different extents. In addition, the cellular defensive system was activated by IRA‐3, as shown in Fig. 4D,E. The activity of a representative antioxidative enzyme, SOD, was decreased by oxidative stress and rescued by IRA‐3. Furthermore, the level of an endogenous antioxidant, GSH, was significantly up‐regulated by IRA‐3.

### Activation of the Keap‐Nrf2‐ARE pathway by IRA‐3

**Fig. 5:**
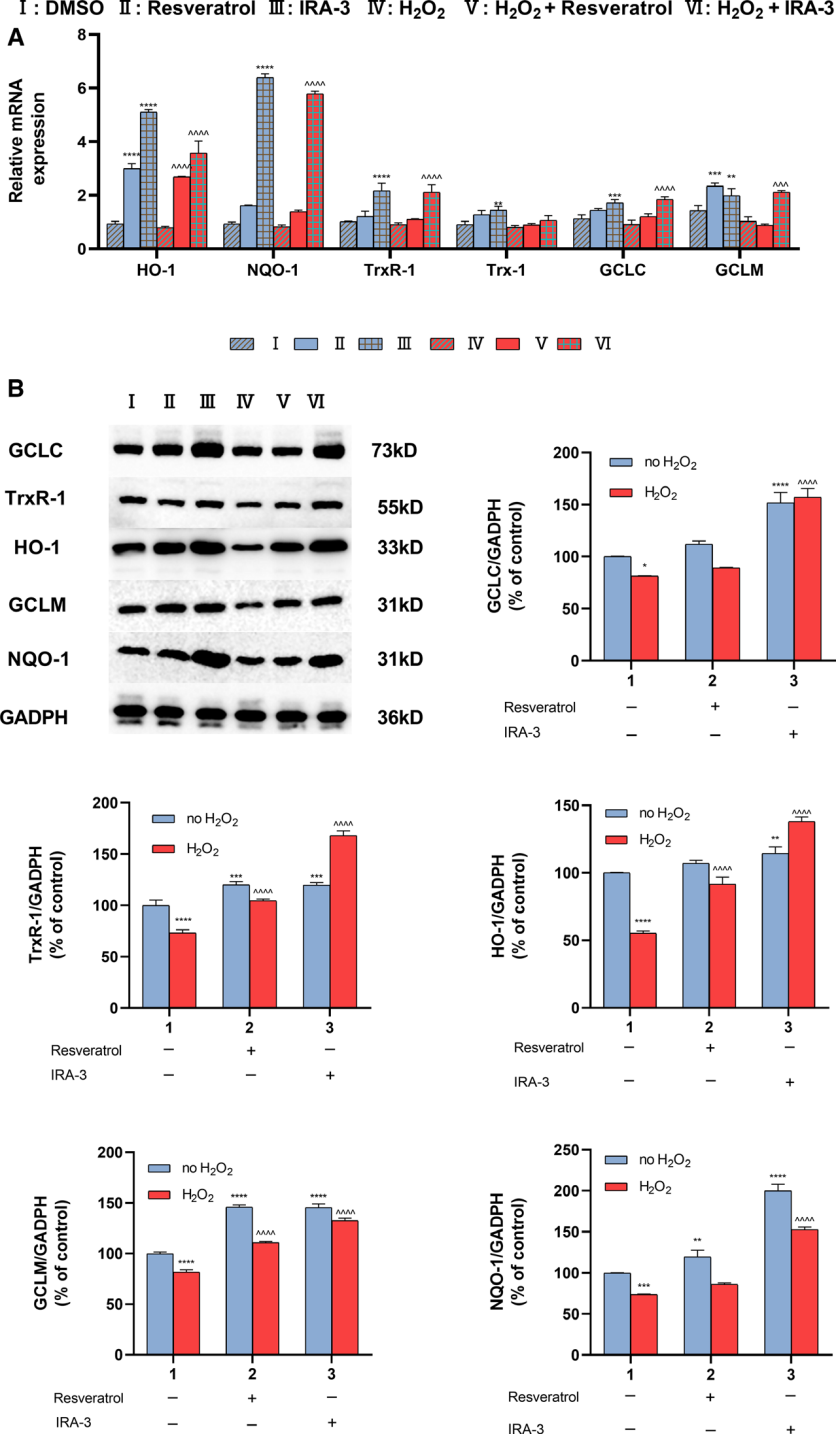
Effects of IRA‐3 on the activation of Nrf2 in normal and model PC12 cells. Cells were treated with 0 or 100 μm H_2_O_2_ for 6 h, and then 50 μm of resveratrol or IRA‐3 was added. After incubation for 24 h, the expression of mRNAs associated with Nrf2 was assessed by a quantitative RT‐PCR (A). Expression of Nrf2‐related proteins was evaluated by western blotting (B). The results are presented as the mean ± SD (*n* = 3). *P < 0.05; **P < 0.01; ***P < 0.001; ****P < 0.0001 vs. the control group. ^^^^^ *P* < 0.001; ^^^^^^ *P* < 0.0001 vs. the H_2_O_2_ treatment. Data were analyzed using two‐way analysis of variance.

The Keap‐Nrf2‐ARE pathway is one of the most important pathways in the cellular defensive system for combating oxidative stress. In the present study, the expression of Nrf2‐driven antioxidative genes was determined with a RT‐PCR. As shown in Fig. 5A, the expression levels of HO‐1, NQO‐1, TrxR‐1, Trx‐1, GCLC and GCLM exhibited similar patterns. The incubation of either normal cells or H_2_O_2_‐treated cells with IRA‐3 resulted in significant increases in the expression of these genes. Furthermore, IRA‐3 exhibited enhanced activity compared to resveratrol with respect to up‐regulating all six genes. The expression of the respective proteins was then examined by western blotting (Fig. 5B). The expression of antioxidative enzymes was significantly up‐regulated after treatment with IRA‐3. In particular, the expression of HO‐1 and NQO‐1 was reduced to approximately 60% of the control under H_2_O_2_ damage but was rescued and enhanced by treatment with IRA‐3, increasing to over 145% of the control. Of note, several studies have suggested that Nrf2 activation was promoted under ROS stimulation. However, our results showed that pretreatment of PC12 cells with H_2_O_2_ slightly decreased the levels of downstream proteins of Nrf2. This phenomenon might be attributed to the different stages of oxidative stress stimulation [32]. In the present study, the analysis was carried out at late oxidative stress state, which was after the cells pretreated with 100 μm H_2_O_2_ for 6 h (pretreated with H_2_O_2_) + 24 h (after same volume of serum‐containing medium with 0.1% dimethylsulfoxide was added). In addition, other pathways also possiblly affect the levels of Nrf2 and related proteins. Glycogen synthase kinase‐3 beta is an upstream factors of Nrf2 and it has also been demonstrated that H_2_O_2_ can induce the activity of glycogen synthase kinase‐3 beta and thereby promote the degradation of Nrf2 pathway cullin1/βTRCP [33].

## Conclusions

In summary, previously reported IRAs were evaluated for their neuroprotective effects against ROS in PC12 cell models. IRA‐3 was selected as the most potent compound, being able to rescue PC12 cells from H_2_O_2_‐induced oxidative damage and achieving a recovery percentage of 60.4% at 50 μm. The cytoprotective effect of IRA‐3 was much stronger than that of resveratrol or the positive control. The present findings, together with our previous reports, indicate that the neuroprotective effect of IRA‐3 is achieved via multiple routes, including the direct scavenging of ROS, the rescue of endogenous antioxidants and the activation of the Keap1‐Nrf2‐ARE pathway. Our results demonstrate the potential of IRAs as simple but effective and safe neuroprotective drug leads.

## Materials and methods

### Cell culture and oxidative stress

PC12 cells (rat pheochromocytoma cell lines) were obtained from the Cell Bank of Type Culture Collection of Chinese Academy of Sciences (Shanghai, China). Cells were cultivated in RPMI Medium 1640 (61870036; Gibco, Waltham, MA, USA) supplemented with 10% (v/v) heat‐inactivated horse serum (Beijing Yuanheng Shengma Biology Technology Research Institute, Beijing, China) and 5% (v/v) fetal bovine serum (#10091148; Gibco) containing penicillin–streptomycin (100×, Q6532; Shanghai Macklin Biochemical, Shanghai, China) and incubated at 37 °C with 5% CO_2_. Culture medium was replaced by fresh medium every 2 days, and cells were passaged upon reaching 80% confluency. Oxidative stress was induced by incubating cells with diverse concentrations of H_2_O_2_ in serum‐free medium (10–400 μm) for 6 h at 37 °C.

### Preparation of IRAs and cell treatments

Imine resveratrol analogs were synthesized as described previously [18, 19] and the characterization data are provided in Appendix S1. In the biological assays, IRAs were dissolved in serum‐containing medium with 0.1% dimethylsulfoxide. After treatment with 100 μm H_2_O_2_ for 6 h, the cells in treatment groups were then incubated in presence of IRAs, edaravone, *t*‐butylhydroquinone (TBHQ) or resveratrol (25, 50, 100 μm) for 24 h. The cells in H_2_O_2_ group were treated in the same way without addition of drugs. For cells in control group, no H_2_O_2_ or drug was added. The corresponding assays were then carried out.

### Cell viability assay and calculation of cell recovery percentage

A cell counting kit‐8 (CCK‐8) assay (ZP328‐3; Beijing Zoman Biotechnology, Beijing, China) was used to measure cell viability. In brief, 1 × 10^4^ cells were seeded into each well of 96‐well plates and cultured for 24 h. After appropriate treatments, the cells incubated with 10% CCK‐8 serum‐free solution, and cells were incubated at 37 °C for an additional 3 h. Absorbance was measured at 450 nm using a microplate reader (Molecular Devices, San Jose, CA, USA). Assays were repeated at least three times.

The cell recovery percentage is defined as:

Cell recovery percentage (%) = (*V*_exp_ − *V*_model_)/(*V*_control_ − *V*_model_) × 100%

where *V*_exp_ is the cell viability of the experimental group, *V*_model_ is the cell viability of the H_2_O_2_‐treated group and *V*_control_ is the cell viability of the control group.

### Calcein AM release assay

The cell viability assay was conducted using a Calcein AM Cell Viability Assay Kit (C2013M; Beyotime Biotechnology, Shanghai, China). After appropriate treatments, cells were incubated with test working fluid at 37 °C for 30 min. Absorbance was measured using a microplate reader. In addition, the cells were double‐stained with Calcein AM and propidium iodide (PI) (ST511; Beyotime Biotechnology) in the case of 50 μm administration. Cell images were recorded using inverted fluorescence microscopy under the green and red channels.

### Detection of ROS

Intracellular ROS generation was monitored using 2′,7′‐dichlorofuorescin diacetate (DCFH‐DA) (S0033; Beyotime Biotechnology). After treatments, cells were incubated with 1 μL of DCFH‐DA (10 μm) at 37 °C for 30 min in the dark. Then, cells were harvested, washed three times with phosphate‐bufffered saline (PBS), resuspended in PBS and then detected by using flow cytometry (Becton Dickinson, Frankin Lakes, NJ, USA).

### Assays for antioxidant enzyme activity

The activity of superoxide dismutase (SOD), GSH and MDA content was determined using the corresponding assay kits (S0109, S0052, S0131; Beyotime Biotechnology). PC12 cells (1 × 10^5^ cells·mL^−1^) were seeded in six‐well plates and then 100 μm H_2_O_2_ was added, with treatment for 6 h. Next, the cells were cultured with or without 50 μm IRA‐3 and resveratrol for 24 h. Then PC12 cells were washed with PBS, and the cells were collected and lysed with cell lysis buffer for western blotting and immunoprecipitation (P0013; Beyotime Biotechnology) treatment. After centrifugation at 14 000 ***g*** for 5 min at 4 °C, the supernatant fractions were collected. The assays were conducted according to the kit instructions using a Microplate Reader.

### Measurement of lactate dehydrogenase (LDH)

The assessment of H_2_O_2_‐induced cell injury was performed by detection of LDH released into the medium. For this experiment, PC12 cells were seeded in 96‐well plates (1 × 10^4^ cells per well) for 24 h and underwent various treatments. The leakage of LDH was evaluated by measuring its activity in the culture medium using the LDH cytotoxicity assay kit in accordance with the manufacturer’s instructions (C0017; Beyotime Biotechnology). Absorbance was determined at 490 nm using a microplate reader.

### Detection of intracellular NO

Cells (2 × 10^5^ cells per well) were seeded into six‐well plates and given serum‐containing medium for 24 h with diverse treatments. NO levels were determined by reaction with Griess reagent in accordance with the manufacturer’s instructions (S0021; Beyotime Biotechnology).

### Quantitative reverse transcriptase‐PCR (RT‐PCR)

Total RNA was extracted from PC12 cells using Trizol Reagent (A33252; Invitrogen, Carlsbad, CA, USA). The RNA concentration was measured with 5X All‐In‐One RT MasterMix (G490; Applied Biological Materials Inc., Richmond, Canada). Extracts were treated with RNase‐free DNase to remove any residual genomic DNA. Subsequently, reverse transcription was used to obtain cDNA. Quantitative RT‐PCR was carried out on an ABI 7500 Real‐time PCR System (Applied Biosystems, Waltham, MA, USA). After 10 min of pre‐incubation at 95 °C, PCR was performed according to the program: 40 cycles of denaturation at 95 °C for 10 s, annealing at 60 °C for 34 s and elongation at 72 °C for 20 s. The expression levels were normalized to GADPH. Relative levels of mRNA were analyzed using the primers: heme oxygenase‐1 (HO‐1): 5′‐GCCCTGGAAGAGGAGATAGAG‐3′ (forward) and 5′‐TAGTGCTGTGTGGCTGGTGT‐3′ (reverse); thioredoxin‐1 (Trx‐1): 5′‐CCTTCTTTCATTCCCTCTGTGAC‐3′ (forward) and 5′‐CCCAACCTTTTGACCCTTTTTAT‐3’ (reverse); thioredoxin reductase 1 (TrxR‐1): 5′‐CGTCCTATGTCGCCTTGGAA‐3′ (forward) and 5′‐TTTGTTGGCCATGTCCTGGT‐3′ (reverse); NAD(P)H quinone dehydrogenase (NQO‐1): 5′‐TCACCACTCTACTTTGCTCCAA‐3′ (forward) and 5′‐TTTTCTGCTCCTCTTGAACCTC‐3′ (reverse); glutamate‐cysteine ligase catalytic subunit (GCLC): 5′‐CAAGGACAAGAACACACCATCT‐3′ (forward) and 5′‐CAGCACTCAAAGCCATAACAAT‐3′ (reverse); glutamate‐cysteine ligase modifier subunit (GCLM): 5′‐GGCACAGGTAAAACCCAATAGT‐3′ (forward) and 5′‐TTCAATGTCAGGGATGCTTTCT‐3′ (reverse); GADPH: 5′‐ CATCACCATCTTCCAGGAGCG‐3′ (forward) and 5′‐ TGACCTTGCCCACAGCCTTG‐3′ (reverse).

Relative mRNA expression values were calculated by the 2^−ΔΔCt^ method.

### Western blot analysis

PC12 cells were incubated in six‐well plates at a density of 2 × 10^5^ and incubated for 24 h at 37 °C. After suitable treatments, the cells were collected and lysed with ice‐cold RIPA lysis buffer. Equal amounts of protein samples (80 μg) were separated on a 10% SDS/PAGE gel and transferred electrophoretically onto nitrocellulose membrane. Subsequently, the membranes were blocked with 5% skim milk and incubated overnight at 4 °C with primary antibody: anti‐HO‐1 (ab68477; Abcam Biotechnology, Cambridge, MA, USA), anti‐GCLC (ab190685; Abcam Biotechnology), anti‐GCLM(ab126704; Abcam Biotechnology), anti‐NQO‐1 (ab124954; Abcam Biotechnology), anti‐TrxR‐1 (ab124954; Abcam Biotechnology) and anti‐β‐actin (AP0060; Bioworld Technology, Bloomington, MN, USA). After being washed three times with 1× Tis‐buffered saline‐Tween 20, the membrane was incubated with anti‐rabbit IgG for 1 h at room temperature. Proteins were visualized by exposure in a ChemiDoc XRS + system (Bio‐Rad, Hercules, CA, USA). Band density was quantified by imagej (National Institute of Health, Bethesda, MD, USA).

### Hoechst 33342 staining assay

Hoechst 33342 is a cell membrane permeable dye. It binds to DNA and gives a strong blue fluorescence. PC12 cells were seeded in a 12‐well plate (1 × 10^5^ cells per well) for 24 h and then treated with H_2_O_2_ (100 μm) for 6 h. After replacing the culture medium with the fresh one containing IRA‐3 (50 μm) or Resveratrol (50 μm) and growth for 24 h, the cells were stained with Hoechst 33342 (5 µg·mL^−1^; C1026; Beyotime Biotechnology) in fresh medium without fetal bovine serum for 15 min at 37 °C. Cell images were recorded via inverted fluorescence microscopy under the blue channel.

### ELISA

Levels of apoptosis parameters including caspase‐3, B cell lymphoma‐2 (Bcl‐2) and Bcl‐2‐associated X protein (Bax) were measured by applying relevant test kits (MB‐7014A, MB‐1845A, MB‐6629A; Jiangsu Mei Biao Biological Technology, Jiangsu, China). Cells were divided into five groups: control (serum‐containing medium with 0.1% dimethylsulfoxide), H_2_O_2_ (100 μm) model, IRA‐3 (50 μm), Resveratrol (50 μm), H_2_O_2_ (100 μm) + IRA‐3 (50 μm) and H_2_O_2_ (100 μm) + Resveratrol (50 μm) group. Detection was conducted in accordance with the manufacturer's instructions. Absorbance was measured at 450 nm using a microplate reader.

### Flow cytometry analysis of cell apoptosis

Cells were seeded in six‐cell plate and incubated overnight at 37 °C and then incubated with H_2_O_2_ (100 μm) for 6 h followed by 50 μm resveratrol or IRA‐3 treatment for 24 h. After that, the cells were stained with annexin V and PI using an annexin V apoptosis kit (C1062M; Beyotime Biotechnology) in accordance with the manufacturer’s instructions. The stained cells were then analyzed by flow cytometer (FC500; Beckman Colter, Indianapolis, IN, USA) to distinguish among viable (annexin V^−^/PI^−^), early apoptotic (annexin V^+^/PI^−^), late apoptotic (annexin V^+^/PI^+^) cells and necrotic cells (annexin V^−^/PI^+^).

### Statistical analysis

Data were presented as the mean ± SD of three independent experiments. Statistical differences between groups were determined by unpaired one‐way or two‐way analysis of variance for multiple comparisons in graphpad pro (GraphPad Software Inc., San Diego, CA, USA). *P* < 0.05 was considered statistically significant.

## Conflict of interests

The authors declare that they have no conflicts of interest.

## Author contributions

YZ, HW and CL designed the experiments. YZ, ZW, JY and CL performed the experiments. YZ and CL wrote the paper. JY and YH analyzed the data. HW and CL supervised the research and revised the manuscript.

## Supporting information


**Appendix S1**. Characterization of IRAs.

## Data Availability

Data are available from the corresponding author upon reasonable request.
