# Supplementary material for: Analogs of imine resveratrol alleviate oxidative stress‐induced neurotoxicity in PC12 cells via activation of Nrf2
Source: FEBS Open Bio. 2021 Jul 2;11(8):2127–38. doi: 10.1002/2211-5463.13209 (PMC8329772; doi:10.1002/2211-5463.13209)
Supplement: Supplementary file 1 — Appendix S1. Characterization of IRAs. [file FEB4-11-2127-s001.pdf]

Analogs of imine resveratrol alleviate oxidative stress-induced neurotoxicity in PC12 cells via activation of Nrf2

Yin Zhang,[1] Zhixiong Wang, [1] Jiehong Yang, [1] Yu He, [1]Haitong Wan,[1] Chang Li*,[1]*

[1] Zhejiang Chinese Medical University, Hangzhou, 310057, PR China

* Corresponding Authors

* Chang Li, lichang@zju.edu.cn; Tel./Fax: +86 571 86613716

* Haitong Wan, whtong@126.com; Tel./Fax: +86 571 86613716

Supporting Information

Characterization of imine resveratrol analogs

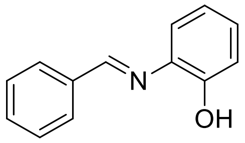

IRA-1: ^{1}H NMR (500 MHz, $CDCl_3$): δ 6.93 (t, J = 7.55 Hz, 1H), 7.05 (d, J = 7.95 Hz, 1H), 7.23 (t, J = 7.60 Hz, 1H), 7.32 (d, J = 7.85 Hz, 1H), 7.50-7.52 (m, 3H), 7.93-7.94 (m, 2H), 8.71 (s, 1H); ^{13}C NMR (125 MHz, $CDCl_3$): δ 115.2, 116.1, 120.3, 129.0, 129.1, 129.2, 131.9, 135.7, 136.0, 152.5, 136.0, 152.5, 157.3; HR-ESI-MS: *m/z* $[M-H]^-$ 196.0766 (calcd for $C_{13}H_{12}NO$, 196.0775).

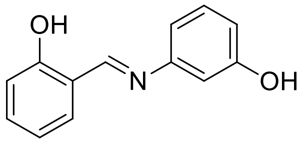

IRA-2: ^{1}H NMR (500 MHz, DMSO-d_6): δ 6.73-6.84 (m, 3H), 6.95-6.98 (m, 2H), 7.24 (t, J = 7.95 Hz, 1H), 7.39-7.42 (m, 1H), 7.64 (dd, J = 7.55, 1.35 Hz, 1H), 8.89 (s, 1H), 9.68 (s, 1H), 13.15 (s, 1H); ^{13}C NMR (125 MHz, DMSO-d_6): δ 108.7,112.6, 114.6, 117.1, 119.6, 119.8, 130.7, 133.1, 133.8, 149.8, 158.8, 160.8, 163.7; HR-ESI-MS: *m/z* $[M+H]^+$ 214.0868 (calcd for $C_{13}H_{12}NO_2$, 214.0863).

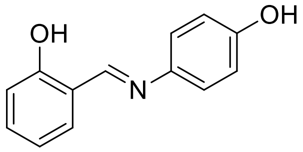

IRA-3: ^{1}H NMR (500 MHz, DMSO-d_6): δ 6.84 (d, J = 8.7 Hz, 2H), 6.92-6.97 (m, 2H), 7.31 (s, 1H), 7.32 (s, 1H), 7.35-7.38 (m, 1H), 7.59 (dd, J = 7.65, 1.4 Hz, 1H), 8.90 (s, 1H),9.67 (s, 1H), 13.42 (s, 1H); ^{13}C NMR (125 MHz, DMSO-d_6): δ 116.9, 117.4, 119.9, 120.4, 123.6, 133.2, 133.5, 140.2, 157.9, 161.1, 161.2; HR-ESI-MS: *m/z* $[M+H]^+$ 214.0859 (calcd for $C_{13}H_{12}NO_2$, 214.0863).

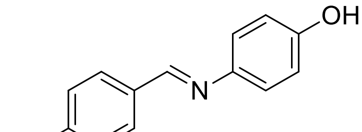

IRA-4: ^{1}H NMR (500 MHz, DMSO-d_6): δ 6.79 (d, J = 5.6 Hz, 2H), 6.87 (d, J = 5.75 Hz, 2H), 7.12 (d, J = 5.75 Hz, 2H), 7.73 (d, J = 5.75 Hz, 2H), 8.44 (s, 1H), 9.43 (s, 1H), 10.05 (s, 3H); ^{13}C NMR (125 MHz, DMSO-d_6): δ 116.6, 116.7, 123.2, 129.0, 131.3, 144.3, 156.7, 157.9, 161.2; HR-ESI-MS: *m/z* $[M+H]^+$ 214.0864 (calcd for $C_{13}H_{12}NO_2$, 214.0863).

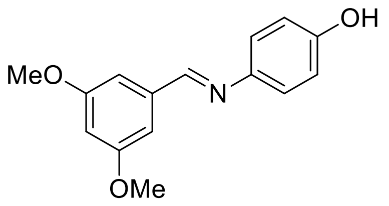

IRA-5: ^{1}H NMR (500 MHz, DMSO-d_6): δ 3.80 (s, 6H), 6.61 (s, 1H), 6.89 (d, J = 7.85 Hz, 2H), 7.06 (s, 2H), 7.19 (d, J = 7.85 Hz, 2H), 8.53 (s,1H), 9.53 (s, 1H); ^{13}C NMR (125 MHz, DMSO): δ 56.4, 104.1, 106.9, 116.7, 123.6, 139.5, 143.4, 157.4, 158.1, 161.6; HR-ESI-MS: *m/z* $[M+H]^+$ 258.1124 (calcd for $C_{15}H_{16}NO_3$, 258.1125).

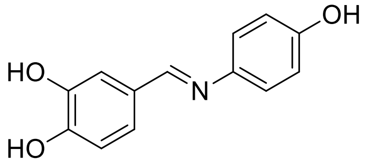

IRA-6: ^{1}H NMR (500 MHz, DMSO-d_6): δ 6.76 (s, J = 5.0Hz, 2H), 6.81 (d, J = 5.0 Hz, 1H), 7.09-7.14 (m, 3H), 7.36 (s, 1H), 8.35 (s, 1H), 9.36 (br, 1H); ^{13}C NMR (125 MHz, DMSO-d_6): δ 114.4, 115.9, 116.1, 122.4, 128.9, 143.7, 146.1, 149.1, 156.1, 157.5; HR-ESI-MS: *m/z* $[M+H]^+$ 230.0744 (calcd for $C_{13}H_{12}NO_3$, 230.0747).

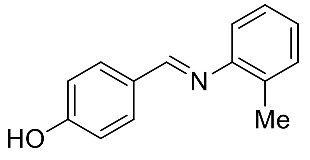

IRA-7: ^{1}H NMR (500 MHz, DMSO-d_6): δ 2.32 (s, 3H), 6.90 (d, J = 5.0 Hz, 2H), 7.01 (m, 3H), 7.25 (t, J = 10.0 Hz, 1H), 7.77 (d, J = 10.0 Hz, 2H), 8.43 (s, 1H), 10.18 (br, 1H); ^{13}C NMR (125MHz, DMSO-d_6): δ 22.2, 116.9, 119.2, 122.6, 127.2, 130.2, 131.8, 139.6, 153.2, 160.9, 161.8. ESI-MS: *m/z* $[M+H]^+$ 212.1008 (calcd for $C_{14}H_{14}NO$, 212.1005).

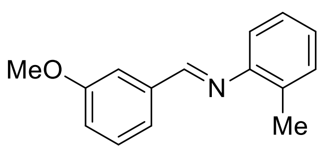

IRA-8: ^{1}H NMR (500 MHz, $CDCl_3$): δ 1.47 (s, 3H), 2.88 (s, 3H), 5.98 (d, J = 7.65 Hz, 1H), 6.08 (dd, J = 5.75, 2.35 Hz, 1H), 6.20 (t, J = 7.35 Hz, 1H), 6.28 (dd, J = 7.15, 4.90 Hz, 2H), 6.41 (t, J = 7.81 Hz, 1H), 6.49 (d, J = 7.45 Hz, 1H), 6.63 (s, 1H), 7.35 (s, 1H); ^{13}C NMR (125 MHz, $CDCl_3$): δ 17.9, 55.3, 112.2, 117.8, 117.8, 122.2, 125.8, 126.8, 129.7, 130.3, 131.9, 137.9, 151.0, 159.3, 160.0; HR-ESI-MS: *m/z* $[M+H]^+$ 226.1228 (calcd for $C_{15}H_{16}NO$, 226.1226).

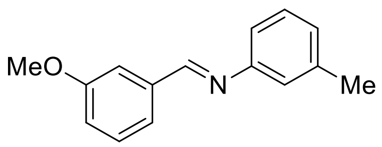

IRA-9: ^{1}H NMR (500 MHz, $CDCl_3$): δ 2.41 (s, 3H), 3.90 (s, 3H), 7.04-7.08 (m, 4H), 7.30 (t, J = 10.0 Hz, 1H), 7.37-7.43 (m, 2H), 7.54 (s, 1H), 8.44 (s, 1H); ^{13}C NMR (125 MHz, $CDCl_3$): δ 21.6, 55.6, 112.0, 118.1, 118.5, 121.8, 122.6, 127.0, 129.2, 129.9, 137.9, 152.1, 160.2, 160.3; HR-ESI-MS: *m/z* $[M+H]^+$ 226.1224 (calcd for $C_{15}H_{16}NO$, 226.1226).

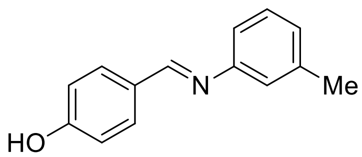

IRA-10: ^{1}H NMR (500 MHz, DMSO-d_6): δ 2.32 (s, 3H), 6.88 (d, J = 8.45 Hz, 2H), 6.97-7.01 (m, 3H), 7.25 (t, J = 7.80 Hz, 1H), 7.76 (d, J = 8.50 Hz, 2H), 8.43 (s, 1H); ^{13}C NMR (125 MHz, DMSO-d_6): δ 22.0, 116.6, 119.0, 122.4, 127.0, 128.5, 129.9, 131.6, 139.4, 153.0, 160.7, 161.6; HR-ESI-MS: *m/z* $[M+H]^+$ 212.1071 (calcd for $C_{14}H_{14}NO$, 212.1070).

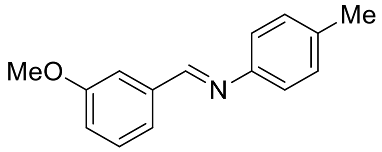

IRA-11: ^{1}H NMR (500 MHz, $CDCl_3$): δ 2.17 (s, 3H), 3.64 (s, 3H), 6.86 (dd, J = 7.90, 1.70 Hz, 1H), 6.97-7.01 (m, 4H), 7.15 (t, J = 7.65 Hz, 1H), 7.20 (d, J = 7.40 Hz, 1H), 7.35 (s, 1H), 8.21 (s, 1H); ^{13}C NMR (125 MHz, $CDCl_3$): δ 21.0, 55.3, 111.8, 118.1, 120.9, 122.3, 129.7, 129.8, 135.8, 137.8, 149.3, 159.5, 160.0; HR-ESI-MS: *m/z* $[M+H]^+$ 226.1232 (calcd for $C_{15}H_{16}NO$, 226.1226).

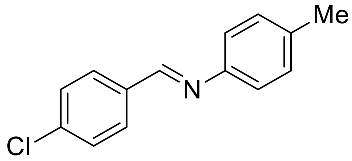

IRA-12: ^{1}H NMR (500 MHz, $CDCl_3$): δ 2.40 (s, 3H), 7.16 (d, J = 10.0 Hz, 2H), 7.22 (d, J = 10.0 Hz, 2H), 7.44 (d, J = 5.0 Hz, 2H), 7.84 (d, J = 10.0 Hz, 2H), 8.43 (s, 1H); ^{13}C NMR (125 MHz, $CDCl_3$): δ 21.1, 120.9, 129.1, 129.9, 134.9, 136.2, 137.2, 149.1, 158.0; HR-ESI-MS: *m/z* $[M+H]^+$ 230.0662 (calcd for $C_{14}H_{13}NCl$, 230.0666).

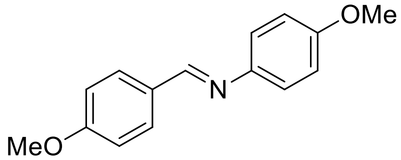

IRA-13: ^{1}H NMR (500 MHz, $CDCl_3$): δ 3.84 (s, 3H), 3.88 (s, 3H), 6.93 (d, J = 8.85 Hz,

2H), 6.99 (d, J = 8.70 Hz, 2H), 7.22 (d, J = 8.80 Hz, 2H), 7.84 (d, J = 8.70 Hz, 2H), 8.41 (s, 1H); ^{13}C NMR (125 MHz, $CDCl_3$): δ 55.6, 55.7, 114.4, 114.6, 122.3, 129.7, 130.5, 145.5, 158.1, 158.1, 162.2; HR-ESI-MS: *m/z* $[M+H]^+$ 242.1175 (cacld for $C_{15}H_{16}NO_2$, 242.1176).

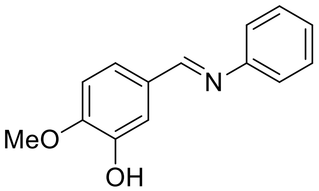

IRA-14: ^{1}H NMR (500 MHz, $CDCl_3$): δ 3.70 (s, 3H, -OCH_3), 6.76 (s, 1H, Ar-H), 6.82 (d, J = 6.56Hz, 2H, Ar-H), 6.89 (d, J = 6.5Hz, 1H, Ar-H), 7.04 (s, 1H, Ar-H), 7.10 (d, J = 5.85Hz, 1H, Ar-H), 7.24 (br, 1H, -OH), 7.69 (d, J = 6.45Hz ,2H, Ar-H), 8.42 (s, 1H, CH=N); ^{13}C NMR (125 MHz, $CDCl_3$): δ 55.6, 114.4, 115.0, 116.0, 120.2, 128.4, 129.0, 130.7, 136.1, 152.2, 156.8, 162.6; HR-ESI-MS: *m/z* $[M+H]^+$ 228.1005 (calcd for $C_{14}H_{14}NO_2$, 228.1019).
